# Optimal Sensor Placement for Modal-Based Health Monitoring of a Composite Structure

**DOI:** 10.3390/s22103867

**Published:** 2022-05-19

**Authors:** Sandris Ručevskis, Tomasz Rogala, Andrzej Katunin

**Affiliations:** 1 Institute of Materials and Structures, Riga Technical University, Kipsalas iela 6A, LV-1048 Riga, Latvia; sandris.rucevskis@rtu.lv; 2 Department of Fundamentals of Machinery Design, Faculty of Mechanical Engineering, Silesian University of Technology, Konarskiego 18A, 44-100 Gliwice, Poland; tomasz.rogala@polsl.pl

**Keywords:** optimal sensors placement, structural health monitoring, delamination, composite structure, machine learning

## Abstract

Optimal sensor placement is one of the important issues in monitoring the condition of structures, which has a major influence on monitoring system performance and cost. Due to this, it is still an open problem to find a compromise between these two parameters. In this study, the problem of optimal sensor placement was investigated for a composite plate with simulated internal damage. To solve this problem, different sensor placement methods with different constraint variants were applied. The advantage of the proposed approach is that information for sensor placement was used only from the structure’s healthy state. The results of the calculations according to sensor placement methods were subsets of possible sensor network candidates, which were evaluated using the aggregation of different metrics. The evaluation of selected sensor networks was performed and validated using machine learning techniques and visualized appropriately. Using the proposed approach, it was possible to precisely detect damage based on a limited number of strain sensors and mode shapes taken into consideration, which leads to efficient structural health monitoring with resource savings both in costs and computational time and complexity.

## 1. Introduction

Structural health monitoring (SHM) became an inevitable part of the continuous control of structural conditions in numerous industrial fields, including means of transport, nuclear energy, civil engineering, and many other [1,2,3]. The aim of an SHM system is to collect data from various sensor sources and carry out the necessary processing, including the extraction of key features, damage detection, and prediction. Depending on the type of damage, tested structure, environmental conditions, and several other factors, such systems can appear in various configurations. An additional difficulty in monitoring damage in structures appears when the tested structures are made of composite materials, which are heterogeneous by nature. This also implies the need to consider various types of damage, which do not appear in homogeneous structures, such as delamination, debonding, and other types of interface damage, which are weakly detectable in general [4,5].

In data-driven SHM, damage detection can be regarded as a problem of pattern recognition. Pattern recognition methods can be classified into two learning (or training) schemes: supervised and unsupervised. Their architecture and learning process are mainly dependent on the necessary level of damage identification. The unsupervised learning scheme leads to clustering analysis and some novelty detection methods, such as outlier analysis, kernel density methods, and auto associative neural networks. These methods are focused on the detection of damage using a comparison of features that describe the condition of the intact and damaged structure. In contrast to this, a supervised learning scheme provides the possibility of damage identification, i.e., it makes it possible to detect, localize, and assess the severity of damages. In supervised learning, the training data consist of both a set of feature vectors as well as their known class labels. Thus, the damage localization is performed by an analysis of specific predefined substructures by assigning class labels for the data, which corresponds to damage in a given substructure. In the same way, damage severity is assessed by assigning class labels to data, which correspond to various levels of damage extent. A possible output of such an assessment algorithm within an SHM system can be considered as a discrete class label, which represents Cartesian coordinates of location of damage sites and damage extent represented, e.g., by the loss of stiffness. Examples of algorithms include the following: neural network classifiers, support vector machines, linear and quadratic discriminant analysis, kernel discriminant analysis, and nearest-neighbor classifiers.

One of the oldest and most widespread SHM methods applied in numerous practical applications is the modal-based one. SHM systems based on this method use vibration measurements for structural evaluation, and the common ones include the following: natural frequencies, mode shapes or curvatures, or damping ratios. In the literature, numerous types and configurations of vibration-based SHM systems can be found, including purely experimental studies, numerical model-supported systems, or even systems based on a digital twin concept. An overview of such systems can be found in review papers [6,7,8]. As examples of vibration-based SHM systems, several studies are available in the literature. Numerous applications of vibration-based SHM systems can be found in civil engineering, primarily in the monitoring of bridges [9,10,11,12,13] and other civil structures [14,15,16]. Special attention to vibration-based SHM systems is devoted to aircraft structures. Examples of such systems can be found in [17,18]. The authors of [19] applied the developed SHM system for monitoring an aircraft wing, while Mironov and Doronkin [20] developed a system for monitoring composite helicopter blades based on their operational modal analysis. A useful summary of vibration-based SHM systems in aviation can be found in [1].

SHM systems are usually based on a permanently mounted sensor grid that performs continuous measurements and delivers signals for further evaluation. For a successful assessment of a structural condition, one of the major factors is the location of the sensors, which must ensure the detectability of damage in all physically possible locations [21,22]. This factor refers to one of the most important scientific problems in SHM, namely, optimal sensor placement (OSP), which is a common problem in all the above-referenced applications.

The OSP problem has been well investigated in the available literature and numerous solutions have been proposed with the application of various approaches. Since OSP refers to an acquisition from modal data of a monitored structure, many of these approaches consider modal strain energy (MSE), modal assurance criterion (MAC), or its derivatives as input evaluation data for the evaluation of suitability of sensor location [23,24,25,26,27,28,29,30,31,32,33]. Other popular approaches for sensor location evaluation are based on the determinant of the Fisher information matrix [25,33,34,35,36], effective independence [29,30,37,38], information entropy [39], and several other approaches. More information on such approaches and their comparison can be found in [29,33,40]. In these approaches, the mentioned criteria are minimized or maximized with the use of various evolutionary algorithms, including genetic algorithms [27,30,35,41,42,43,44], particle swarm optimization algorithm [45], simulated annealing algorithm [46], Jaya algorithm [47], Bayes theory approaches [48,49,50], and more. Nevertheless, the OSP problem remains open and the developments on effective algorithms for OSP in various applications are still in progress.

**Figure 1:**
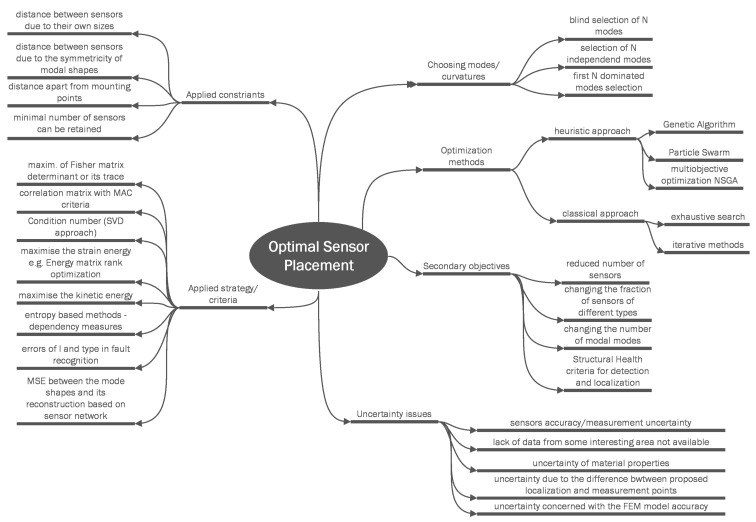
The mind map for solving the OSP problem of a composite plate with damage (based on [19,25,26,27,28,29,31,32,33,34,35,36,37,38,39,40,41,42,43,44,45,46,47,48,49,50,51]).

In the initial stage of research, the authors prepared a mind map (see Figure 1), describing different issues related to the OSP problem. Optimal sensor placement is widely described in the literature; therefore, the authors decided to present a large amount of information on the current state of the art of OSP issues using mind map. The figure presents, among others, selection of modes or curvatures for OSP in different SHM approaches. The figure also presents the secondary OSP objectives based on the reduced number of sensors, the reduced number of modal modes used, and the objectives based on the SHM criteria for the detection and localization of damages. Numerous ideas of the applied constraints and strategies used are also presented in Figure 1. Because uncertainty issues play an important role in OSP, different types of uncertainty aspect presented in different research have been pointed out.

The main drawback of a supervised learning algorithm that can provide a discrete class label for many different damage states is that data (feature vectors) from a tested structure in all of damage states are necessary to perform a training of the algorithm. This solution might be very time consuming and expensive in the case of its experimental implementation. However, it is possible to overcome this problem by using finite element (FE) simulations of a composite structure with a set of different damage states to obtain the necessary training data, which was proposed in this paper. This implies the second goal of this study, namely, ensuring a possibility of effective damage identification regardless of the location of damage, i.e., the sensor network does not have to be established on the basis of data that comes from damage conditions. The OSP for the supervised learning scheme is determined by using dynamic strain data experimentally measured and numerically calculated to derive sensor networks. To obtain an optimal one, many metrics of the performance of the sensor’s networks are used separately, or when used together, they provide different solutions [25,51]. In this paper, an aggregation Hamachart function of t-conorm and t-norm is proposed to incorporate different metrics in the assessment of the sensor network. The results obtained are used in five pre-selected machine learning algorithms within two supervised learning schemes. Based on the obtained results it was possible to indicate which of the applied OSP methods leads to better results in the sense of the accuracy of classifiers.

## 2. FE Model of a Composite Plate

For the training data to be accurate, one must establish a physical-law-based numerical model of structure and calibrate it in terms of features of interest according to the physical model. For this reason, a method based on the mixed numerical experiment technique (MNET) is employed for numerical model calibration. The main advantage of MNET is the possibility of measuring a number of related quantities and deriving the unknown property from the experimental values of these quantities instead of direct measurement of a given property. In this way, a numerical model is used as a substitution of measured quantities, which is based on relations between a given physical property and the measurement results. This leads to a formulation of an inverse problem, which is used for deriving model parameters based on the response of the system to a given input. This problem is solved by minimizing the difference between the responses of the numerical model and the experimental measurements.

### 2.1. CFRP Plate and Its Parameters

**Figure 2:**
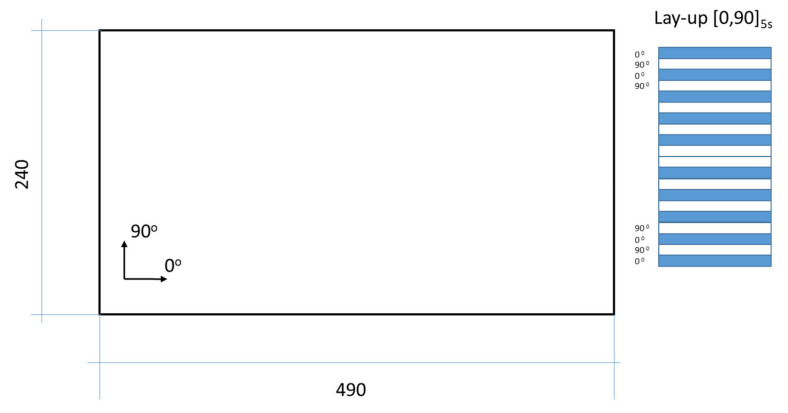
The schematic representation of the CFRP plate.

A carbon fiber-reinforced plastic (CFRP) rectangular plate with dimensions 490 × 240 mm was considered in the study (Figure 2). The plate is manufactured using the hand layup method. Individual prepreg layers are cut from a roll of UNIPREG^®^ Carbon non-crimp fabric 100 g/m^3^ (Unicarbon, Kaunas, Lithuania) accordingly to form a (0/90)_5 s_ laminate lay-up. The assembled laminate is covered with a vacuum bag and sealant tape to create mechanical pressure during its curing cycle. After curing, the hydraulic weighting method was applied to obtain the material density—1545 kg/m^3^. The total thickness (*h*) of the plate was measured to be approximately 1.84 mm, so the individual ply thickness of the laminate is assumed to be 0.092 mm.

The selection of calibration parameters is one of critically important tasks for the effective calibration of the FE model; i.e., changes of particular parameters being tuned should have a considerable effect on the resulting system response. Such a calibration can be performed by using the tuning modal characteristic of the investigated structure, which reveals a high sensitivity to changes both in stiffness and mass properties when damage occurs. Since the mass density and total thickness of the plate have been determined with high accuracy using conventional testing methods, the material properties are selected to be calibrated within the FE model. The calibrated parameters are five engineering constants of a single transversally isotropic layer of CFRP plate: *E*_1_, *E*_2_ = *E*_3_—longitudinal and transverse Young’s moduli; *G*_12_ = *G*_13_—in-plane shear moduli; *G*_23_—out-of-plane shear modulus; *ν*_12_ = *ν*_13_—in-plane Poisson’s ratios.

### 2.2. Experimental Setup for Model Calibration

**Figure 3:**
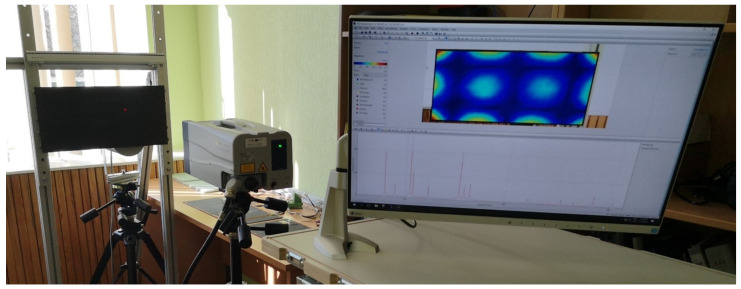
The experimental setup for FE model calibration.

**Figure 4:**
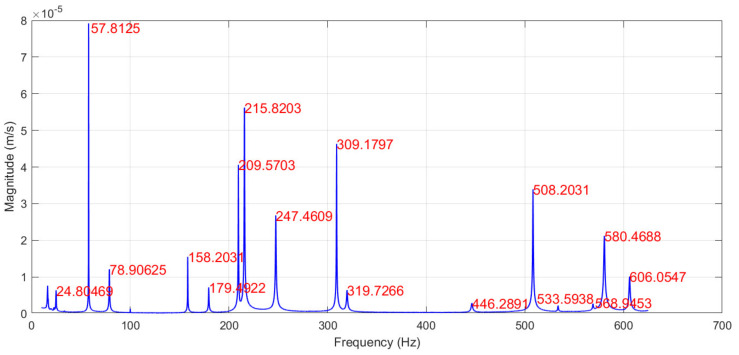
The FRF of the tested CFRP plate from calibration tests.

The modal frequencies and corresponding mode shapes of the tested plate are measured using a Polytec^®^ (Waldbronn, Germany) PSV-500-3D scanning laser vibrometer. The general experiment setup consists of a PSV-I-500/PSV-I-520 optical scanning heads, PSV-F-500 Front-End with 3 digital broadband decoders, signal generator, and data acquisition for reference channels (PSV-500-3D-H); PSV-E-500 junction box, a Bruel & Kjaer (Nærum, Denmark) type 2732 amplifier, and a computer system with installed data acquisition and signal generator board (PSV-500-3D-M); PSV-W-500 Data Management System and PSV software. The plate is excited by a periodic input chirp signal generated by the internal generator with a 0 ÷ 625 Hz bandwidth with a frequency resolution of 0.195 Hz through a loudspeaker Penton CAD10T/EN (Worthing, UK) (see Figure 3) in order to obtain the frequency response function (FRF)—see Figure 4—and resonant frequencies (Table 1). Three averaging cycles were applied to improve the measured response. The specimens were hung using two thin threads that simulate free boundary conditions.

### 2.3. Calibration of Numerical Model

The CFRP plate was modelled using the FE commercial solver ANSYS^®^ (Canonsburg, PE, USA). For FE analyses of thin structures, two classes of shell elements are commonly employed: classical two-dimensional elements and three-dimensional continuum (solid shell) elements. An extensive performance comparison between two of the ANSYS^®^ shell elements, SHELL181 and SOLSH190, can be found in the technical report [52]. The predicted displacements and stresses for various geometries and boundary conditions are compared with exact solutions and simulations using full three-dimensional brick elements, SOLID185. Published results show that classical shell elements and solid shell elements behave in a similar manner for considered problems and the difference in results between shell and brick elements is marginal as far as the bending stress is concerned. The authors conclude that plates modeled with a single layer of solid shell elements are extremely stiff in bending, and they recommend to use at least three elements through the plate thickness for reasonable results.

For the calibration of numerical model of thin composite plates, the most convenient way is to use classical two-dimensional shell elements—one element through the thickness of the plate with homogenized material properties. However, taking into account the delamination problem in the following investigation, employing 3D solid shell elements, SOLSH190, instead of classical shell elements was decided. In general, the SOLSH190 element allows the definition of layers with different properties; thus, the plate could be modeled using one element with twenty layers through the thickness or twenty elements with one layer to be stacked through the thickness to achieve the same effect [52]. Since the delamination is introduced between several layers of the plate, in the present FE model, each of the 20 single transversally isotropic layers is represented with one element through the thickness, resulting in 20 elements in the stacking direction of the layup. The material properties of each layer are defined according to the laminate stacking sequence (see Figure 2). Three integration points through the thickness of the element are adopted in this study as the number have been confirmed to provide stable results [52].

The numerical modal analysis routine with the Lanczos eigensolver was defined to determine the eigenvalues and corresponding eigenvectors of the undamped vibrations of the FFFF (all edges free) composite plate. The modal frequencies and corresponding mode shapes for the first 12 modes are calculated to estimate the deviations between the experimental and numerical frequencies. Obtained modal analysis results are compared with simulations using classical shell elements as well as with experimental results. The comparison results show that 3D solid shell elements allows the determination of dynamic strain profiles with a good accuracy, small uncertainties, and acceptable computational efficiency.

The procedure used for the calibration of the model relies on a parametric model of the plate and the minimization of a functional based on the discrepancy between the measured and calculated natural frequencies [53] as follows:

(1) $$\begin{array}{c} \mathrm{Minimize}\;\Phi \left(x\right)=\sum_{i=1}^{I}w_{i}\frac{{\left(f_{iEXP}^{2}-{\left[f_{iFEM}^{2}\right]}^{2}\right)}^{2}}{f_{iEXP}^{4}}\\ \mathrm{subject}\;\mathrm{to}\;x_{i}^{\min}\leq x_{i}\leq x_{i}^{\max},\;i=1,\ldots ,12,\; \end{array}$$

where $f_{iEXP}^{2}$ and $f_{iFEM}^{2}$ are the eigenfrequencies obtained from experiment and numerical calculations, respectively, $w_{i}$ are the weights for error functionals, and I is the number of considered eigenfrequencies in this study.

**Figure 5:**
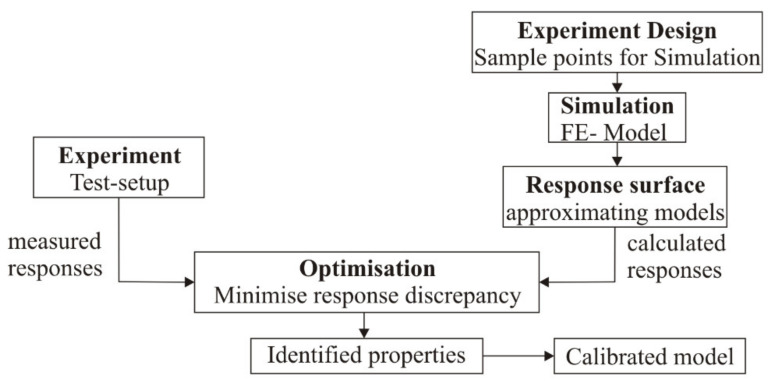
The flowchart of the numerical model calibration method.

The flowchart of the proposed numerical model calibration method is presented in Figure 5.

In the first phase, the physical experiment is performed, and the resulting responses of the structure are recorded. In the second phase, calibration parameters (i.e., design variables), the searching domain, and criteria are selected. This information is then used to define an experiment design task to determine the sample points for numerical simulations. Each sample point in the experiment design represents a unique set of system parameters. In the third phase, numerical simulations are performed only at the sample points of the experiment design. Furthermore, the results of the numerical simulation are used for a definition of response surface approximations that mathematically describe the relationship between the design variables and the response of the investigated structure. In the next phase, the obtained approximations are used to formulate the objective function and the optimization constraints. The procedure is concluded by constrained minimization of a non-linear multivariable function. Using the minimizing functional parameters, calibration parameters were obtained (Table 2).

The verification of the results obtained was based on comparison of the experimentally measured ($f_{i_{EXP}}$) and the numerically calculated eigenfrequencies ($f_{i_{FEM}}$) using the calibrated engineering constants (x*). Residuals ($\Delta_{i}$) were determined using the following expression.

(2) $$\Delta_{i}=\left|\frac{f_{i_{FEM}}\left(x^{*}\right)-f_{i_{EXP}}}{f_{i_{EXP}}}\right|\cdot 100.\;$$

## 3. General Experiments and Definition of OSP

### 3.1. Experimental Modal Analysis

**Figure 6:**
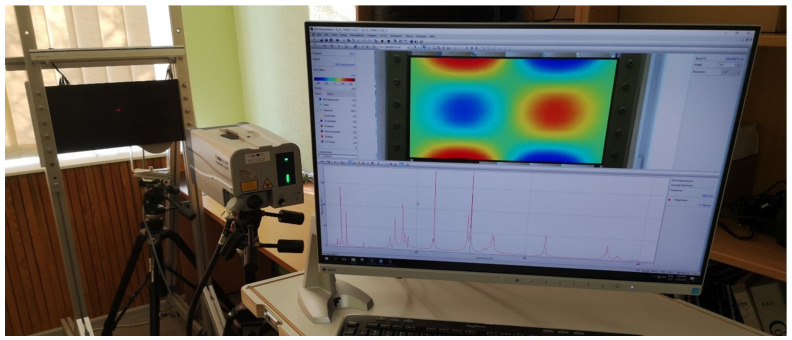
The experimental setup for modal analysis.

Modal analysis was performed using the same CFRP plate described in Section 2.1. The clamped boundary conditions are applied at two edges of the plate (see Figure 6). The distance of 20 mm of edge of the plate is fixed between the frame and solid aluminum bar by means of bolts with the spacing distance of 50 mm. The clamping strength is achieved by applying 20 Nm of fastening torque to the bolts. The resonant frequencies and corresponding mode shapes of the plate are measured using a Polytec^®^ PSV-500-3D scanning laser vibrometer. The general experimental setup consists of a PSV-I-500/PSV-I-520 optical scanning heads, PSV-F-500 Front-End with 3 digital broadband decoders, signal generator and data acquisition for reference channels (PSV-500-3D-H); PSV-E-500 junction box, a Bruel & Kjaer (Nærum, Denmark) type 2732 amplifier, and a computer system with installed data acquisition and signal generator board (PSV-500-3D-M); PSV-W-500 Data Management System and PSV software.

**Figure 7:**
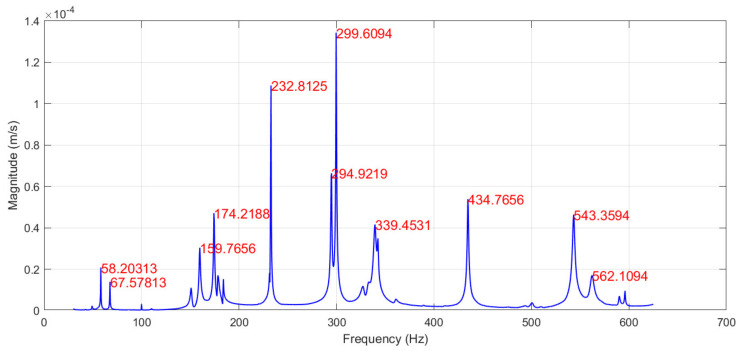
The FRF of the tested CFRP plate from modal analysis.

The scanning grid of 25 × 13 equidistant measurement points is defined. The plate is excited by a periodic input chirp signal generated by the internal generator with a 0 ÷ 625 Hz bandwidth through a loudspeaker to obtain FRF and resonant frequencies (Figure 7). Then, the excitation with a periodic sine-wave signal is applied to the plate with correspondence to the first 7 resonant frequencies to obtain the corresponding mode shapes. To account for the weight of vibration amplitude of a particular mode in the summarized strain plots, the generated vibration power is constant for all resonant frequencies.

Measured transverse displacement values (*w*) of a particular mode are employed to calculate strain values in both principal directions of the plate through the following steps:

1. To obtain useful strain fields, transverse displacement is smoothed using weighted linear least-squares regression analysis with a second-degree polynomial approximation [54]. To fit the original surface as close as possible, a local smoothing process is considered; thus, each smoothed value is determined by neighboring data points defined within the span of 10% of the total number of data points.
2. The largest absolute value of the corresponding mode is obtained for the scaling of the calculated FE transverse displacement values.
3. Strain values on the top surface of the plate in the corresponding directions are calculated by the following expressions.

(3) $$Sx_{\left(i,j\right)}={\left(\frac{\partial^{2}w}{\partial x^{2}}\right)}_{\left(i,j\right)}\cdot \;\frac{1}{2}h;\;Sy_{\left(i,j\right)}={\left(\frac{\partial^{2}w}{\partial y^{2}}\right)}_{\left(i,j\right)}\cdot \;\frac{1}{2}h.$$

4. Strain fields were calculated for the grid of 91 × 25 points to adjust it to the FE model element size of 5 mm.

### 3.2. Numerical Modal Analysis

**Figure 8:**
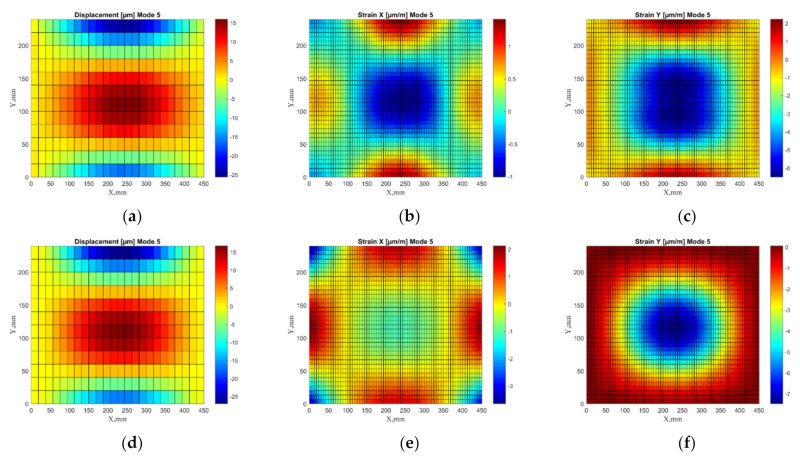
The examples of transverse displacement shapes and strain fields in X- and Y-directions: experimental (**a**–**c**) and numerical (**d**–**f**).

The FE model of the plate is built using the material data (layer stacking, engineering constants) obtained in Section 2.3. A regular mesh of 24 × 12 elements for the plate was generated using the SOLSH190 element type, and the clamped boundary conditions are applied in two regions with a length of 20 mm from the edge of the plate in the same way as in the experimental study (see Section 3.1). Each layer of the composite lamina is represented by one element through the thickness, resulting in 20 elements in the stacking direction of the layup. Modal analysis with the Lanczos eigensolver was applied to determine the first 7 eigenvalues and corresponding eigenvectors for the 25 × 13 nodes of the undamped vibrations of the model. The algorithm for calculating strain values is provided below:

1. The obtained non-dimensional transverse displacement values of the particular mode are normalized with respect to the largest absolute value.
2. Normalized transverse displacement shapes are scaled with respect to the largest absolute value of the corresponding experimentally measured mode. In this way, the weight of vibration amplitude of a particular mode can be accounted for in the summarized strain plots.
3. Strain values in the corresponding directions are calculated by (3).
4. The calculated strain fields are smoothed using regression analysis to obtain a denser grid. For illustration purposes of the approach, a comparison between the experimental and numerical transverse displacement shapes and the derived strain shapes is shown in Figure 8.

### 3.3. Definition of OSP Problem and Selected Methods

The data-driven structural health monitoring system is dependent on the data provided by the distributed sensors. The evaluation of the state of the composite plate, the detection of damage, and the information on its localization depend on the quality of the data recorded by the sensor network. The sensor network is considered to be a limited number of sensors distributed in a determined localization of the composite plate. The goal of the optimal sensor placement problem is to treat the number of sensors and its localization as design variables to obtain the optimal layout, taking into account the different issues presented in Figure 1, such as applied strategies, constraints, etc.

In this study, the minimum number of sensors is considered to minimize the cost of sensor deployment. Another important factor from the point of view of further application of an SHM system is the added mass from sensor deployment, which, in the case of aircraft structures, has a significant impact, even if sensors are lightweight. The influence of deployed sensors on the take-off weight of an aircraft and further economic consequences are discussed in [55]. In addition to that, the number of modes of composite plate is also of interest because sensors should deliver the most relevant dynamic information about the measured structure with limited data processing. As input data, the strains of the selected *i*th-mode shape in X-direction $\epsilon_{x}\left(x,y,i\right)$, and the Y-direction $\epsilon_{y}\left(x,y,i\right)$ are treated as independent input data set, where x and y are coordinates that localize the strains on the plate. Normally, the data take the form of a modal matrix, where the rows contain subsequent values of the strains for X, and then the localizations of Y and columns are related to subsequent shape modes. In the considered case, the columns that represent the modes are ordered according to the highest amplitude of the mode obtained from the experimental frequency response function, so the first column is connected with the highest energy modal shape.

In this study, the following optimal sensor placement methods and their variants are considered:

(1) Method A1. In this method, the input data contain absolute strain values at each grid point for the appropriate numerically calculated modes that are summed without normalization (each shape mode has a different weight).
(2) Method A2. Absolute strain values at each grid point for appropriate numerically calculated modes are normalized with respect to the largest value of the particular mode and then summed (all modes are equally weighted). In methods A1 and A2, the sum of the strain values for each grid point is sorted in a vector in descending order from the highest value. The algorithm searches for the next largest strain value and checks the eligibility for sensor placement taking into account the following constraints:
  a. Distance from the edge of the plate or the edge of the clamp ≥ 5 mm due to the size of the strain sensors;
  b. Distance between any two sensors > 127.5 mm (the mode shapes are symmetric and the summed absolute strain values are the same in four imaginary quadrants of the plate, so the idea is to place the sensors in different quadrants so that they are sensitive to all possible damage locations: $\sqrt{{\left(450/4\right)}^{2}+{\left(240/4\right)}^{2}=127.5}$.
(3) Method A3 is a modified version of an effective independence method proposed in [56], which is still widely applied in many OSP issues [24,27,36,42]. This modification allows one to apply the constraint distance between any two sensors. The pseudocode of this method is as follows.

**Stage I Remove** any candidate sensor points that cannot be measured (e.g., areas under clamps, edges) from the set of possible candidate point localization.

**Stage II Repeat until** the required number of candidate points is obtained:

- **Create** Fisher Information Matrix FIM for all available candidate points;
- **Create** orthogonal projection matrix E;
- **Analyze** the trace of the E matrix and find the index of the minimum value;
- **Store** the history about the removed candidate points;
- **Remove** the candidate point with the minimum value from the trace from the FIM matrix;
- **Create** the set of candidate points.

**Stage III Repeat until** the constraint about the required distance between the candidate points in the set is not met:

- **Find** the candidate points that break the distance constraint with other points;
- **Replace** the previously selected candidate points into other candidate points using the points from the stored history of the previously removed points. For this purpose, use Last Input First Output history sequence queue.

Methods A1, A2, and A3 treat the input data strains $\epsilon_{x}\left(x,y,i\right)$ and $\epsilon_{y}\left(x,y,i\right)$ as independent. It results in two separate sensor networks for x-strain sensors and y-strain sensors. Because in some cases the information from strains in the X direction can be more informative than that from strains in Y direction or vice versa, these constraints have been loosen, and the next three variants of the above A1, A2, and A3 methods are also considered. Methods B1, B2, and B3 refer consequently to previous methods A1, A2, and A3, but the input data of the strains from the *i*th-mode shapes in X-direction $\epsilon_{x}\left(x,y,i\right)$ and the Y-direction $\epsilon_{y}\left(x,y,i\right)$ are treated commonly as one common data set. This results in a sensor network that could have a different fraction of sensors in the X and Y directions.

As already mentioned, the goal of the OSP is not only to determine the distribution of the sensor localization on the composite plate but also to determine the appropriate number of required sensor number and used mode shapes. To evaluate this, different metrics can be applied for this purpose. In the considered study, the following metrics have been applied [24,28,38,51]:

(a) RMS MAC criterion

(4) $$RMS_{MAC}=\frac{1}{\sum \left(i-1\right)}\overset{N}{\underset{i=1}{\sum }}\overset{N}{\underset{j=1\;i\neq j}{\sum }}u{\left(MAC_{i,j}\right)}^{2}\;\;i=1\ldots Nm,\;\;j=1\ldots Nm\;,$$

where i and j are indexes of modes, $u\left(\cdot \right)$ refers to the triangular matrix, $N_{m}$ is the number of modes, and $MAC_{i,j}$ is a matrix of modal assurance criteria defined by:

(5) $$MAC_{i,j}=\frac{{\left(\phi_{i}^{T}\phi_{j}\right)}^{2}}{\left(\phi_{i}^{T}\phi_{i}\right)\left(\phi_{j}^{T}\phi_{j}\right)}\;,$$

where $\phi_{i}\;\mathrm{and}\;\phi_{j}$ are vectors of *i* or *j* modes with strain values of selected sensor networks.

According to the above definitions, RMS_MAC contains the root means square of the off-diagonal elements of the triangular MAC matrix. The improved quality of the sensor network is indicated by low off-diagonal terms.

This criterion can take the form of a relative criterion with the reference point related to the full modal matrix, which means that it can refer to all available candidate points and the maximum number of observable modes:

(b) Fisher matrix determinant:

(6) $$\det\left(FIM\right)=|\det[\Phi^{T}\Phi ]\;|\;,$$

where Φ is the modal matrix of the selected sensor network. This value decreases with the reduction in the number of candidate points, but algorithms based on this criterion usually try to maintain the highest possible value of this determinant for the finally reduced modal matrix for the obtained sensor network:

(c) Condition Number:

(7) $$CN=\frac{svd{\left(\Phi \right)}_{\max}}{svd{\left(\Phi \right)}_{\min}}\;,$$

where *svd* represents the singular value decomposition operation, and $svd{\left(\Phi \right)}_{\max}$ and $svd{\left(\Phi \right)}_{\min}$ represent the maximum and minimum singular values from the modal matrix of considered sensor network. The low value of the condition number indicates a better configuration of the sensor network.

The presented indices allow the performance of different sensor networks to be evaluated, although the high value of one index does not have to be confirmed by other indices: examples of which can be found in [25,51]. Because of this, the following aggregation function based on the Hamacher function [57] has been proposed to support the selection of sensor network configuration:

(8) $$T_{\lambda }\left(a,b\right)=\left\{\begin{array}{cc} 0 & if\;\lambda =a=b=0\\ \frac{ab}{\lambda +\left(1-\lambda \right)\left(a+b-ab\right)} & otherwise \end{array}\right.\;,$$

(9) $$C_{\lambda }\left(a,b\right)=\left\{\begin{array}{cc} 1 & if\;\lambda =a=b=1\\ \frac{a+b-ab-\left(1-\lambda \right)ab}{1-\left(1-\lambda \right)ab} & otherwise \end{array}\right.\;,$$

where $T_{\lambda }$ is a t-norm and $C_{\lambda }$ is a t-conorm of the Hamacher function [57], and a andb correspond, e.g., to RMS MAC and det(FIM), respectively. λ is a parameter that defines how strictly t-norm and t-conorm are decreasing and increasing, respectively.

The aggregation functions require the use of normalized inputs of indices to range from zero to one. Finally, normalized det(FIM) has been applied, so the total distribution of the value of the metrics along the considered range of sensor number and mode number sum to one. Because the optimal values for RMS MAC and CN should be minimalized, the complements of normalized values, e.g., $1-\det\left(FIM\right)$, were used as input values for the aggregation function.

Due to the symmetricity and associativity [57] of the Hamacher function, the number of used metrics can be added to the evaluation of the performance of the candidate sensor networks. In the same manner, other t-norms and t-conorms family functions can be applied for this purpose.

### 3.4. Evaluation and Selection of Sensor Networks

**Figure 9:**
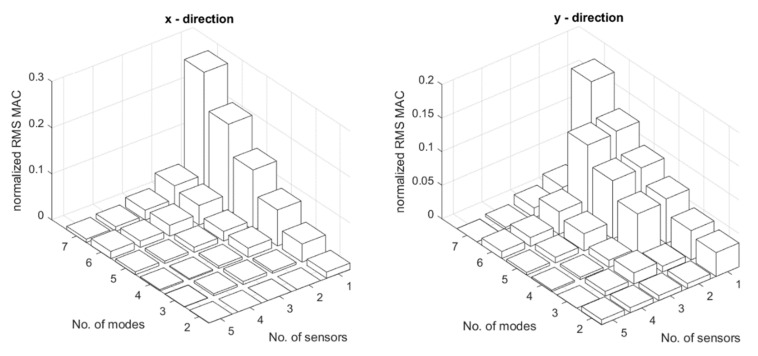
Normalized RMS MAC for the A2 method (left for the *x* sensors and right for the *y* sensors) obtained for the different number of modes and number of sensors.

**Figure 10:**
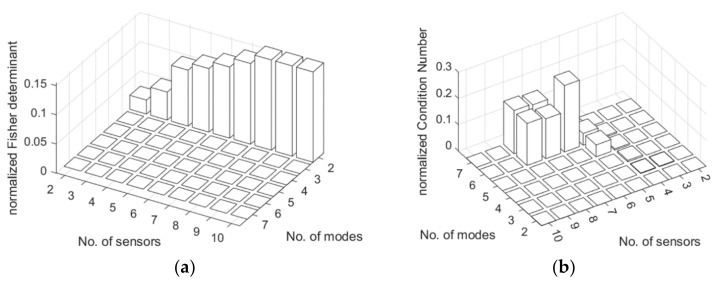

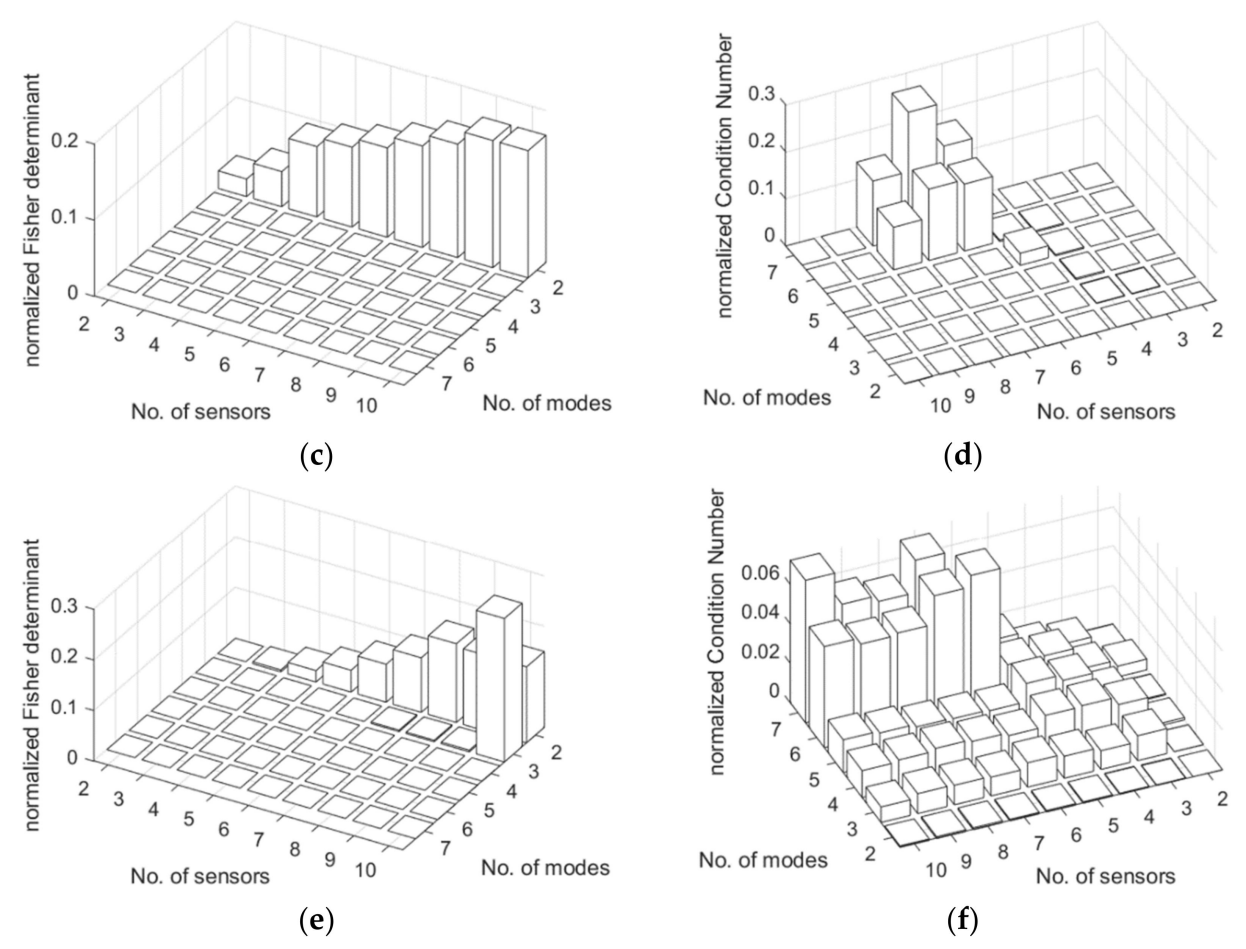
Distribution of normalized Fisher determinant (**a**,**c**,**e**), and distribution of Normalized Conditional number for B1 (**a**,**b**); B2 (**c**,**d**); and B3 (**e**,**f**). Methods obtained for the different number of modes and number of sensors, respectively.

Using the methods presented in Section 3.3 different candidate sensors networks can be obtained. To select appropriate design variables of the sensor networks, the number of sensors and used modes was calculated. This resulted in 251 sensor networks candidates. For methods A1, A2, and A3, the number of sensors changed in a range of 1 to 5 and the number of modes changed from 2 to 7. For methods B1, B2, and B3 the number of modes was changed in the same range as for A type methods. The number of sensors for B1, B2, and B3 changed from 2 to 10, finally providing the opportunity to obtain the same total number of the sensors, as in the case of A1, A2, and A3 methods where the number of sensors in the *x* and *y* directions independently was limited to 5 (and totally to 10). Next, all candidate sensor obtained with different methods were evaluated using different metrics shown in Section 3.3. Figure 9 and Figure 10 show selected distributions of different normalized metrics obtained for different methods.

**Figure 11:**
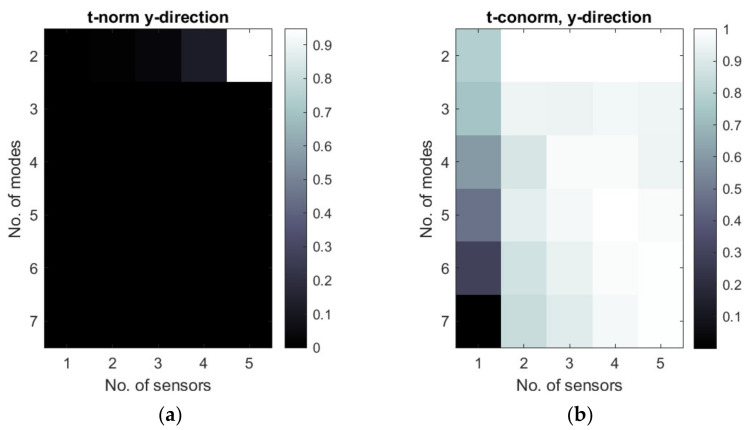
Map of t-norm (**a**) and t-conorm (**b**) values of normalized RMS MAC and Fisher determinant for sensor networks calculated with the A1 method. The number of sensors in y-direction is considered.

**Figure 12:**
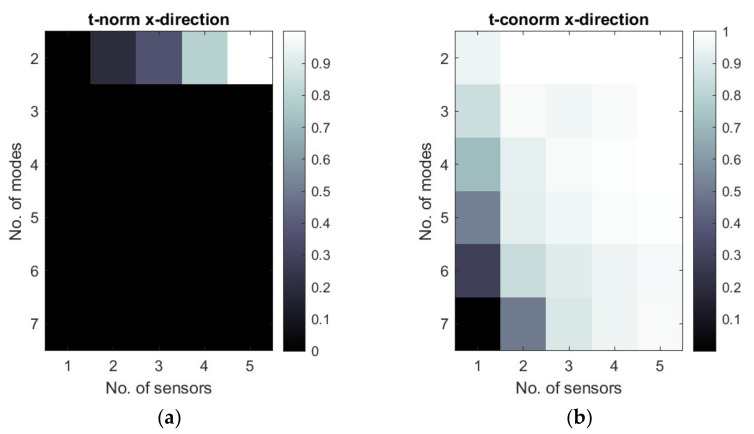
Map of t-norm (**a**) and t-conorm (**b**) values of normalized RMS MAC and Fisher determinant for sensor networks calculated with the A3 method. The number of sensors in *x*-direction is considered.

**Figure 13:**
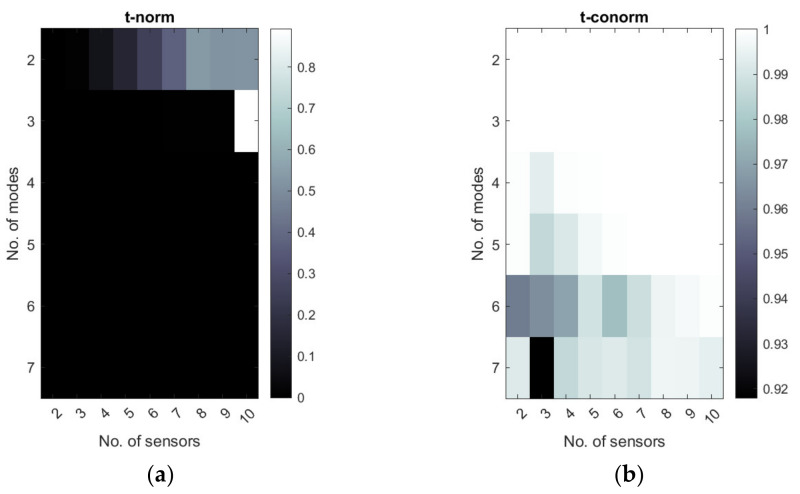
Map of t-norm (**a**) and t-conorm (**b**) values for the B3 method (normalized RMS MAC, Fisher determinant, and Conditional number were used in aggregation).

Figure 11, Figure 12 and Figure 13 show the selected examples of the map of t-norms and t-conorms of the Hamacher aggregation function for different methods and with the application of different normalized metrics number. For example, Figure 11 and Figure 12 show that on the basis of the RMS MAC and Fisher determinant using t-conorms, the appropriate sensors network for the placement of y- sensors obtained using the A1 method can be obtained using 2 dominated modes and 2–5 sensors and also for 5 modes and 4 sensors. Similarly, appropriate sensor networks obtained for the A3 method in y-strain sensors in the sense of the proposed aggregation function can be obtained for 2 modes and 2–5 sensors, as well as for 3–4 modes and 5 sensors. Figure 13 shows that, for method B3, the t-norm based on all 3 metrics can lead to a wider set of allowable sensor networks. The t-conorms presented in Figure 11, Figure 12 and Figure 13 usually suggest solutions with a larger number of sensors, but they are very limited in the number of modes used.

The above presented Figures of maps of t-conorms and t-norms can be helpful in the selection of appropriate parameters of sensor networks for the SHM system. In the considered research study, mainly RMS MAC and Fisher Determinant were used as inputs for the t-norm and t-conorm functions. Next, the set of allowable (mainly for t-conorms) set of allowable sensor networks can be extended/reduced on the basis of Condition Number and associativity of aggregation functions if necessary. The allowable set of sensor networks obtained using the A1, A2, and A3 method is presented in Table 3. This table presents the localization of the individual X and Y strain sensors in the localization together with information on the number of sensors used in the X (sX) and Y directions (sY), and the number of dominant modes (mX and mY) used to establish the considerer sensor network. Similarly, the sensor networks obtained using the B1, B2, and B3 methods are presented in Table 4. In this table, the sensor networks SN#4, SN#5, and SN#6 were obtained using the B1, B2, and B3 method correspondingly. Columns nS and sM present the number of sensors used and the modes used for the considered sensor networks.

Since different metrics of sensors networks can lead to quite a different set of possible sensor networks, the use of the t-norm is not always the right choice, especially if the number of used metrics is large—this may lead to the empty set of solutions. The reason is that the t-norm has properties of the type of conjunctive function. In this case, the t-conorm can lead to a wider set of possible configurations of sensor networks in the sense of the number and modes. Table 5 and Table 6 present selected sensor networks obtained using different methods using t-conorms maps. Because t-conorms sensors networks usually also contain the sensor networks suggested by the t-norm, the choice from the set of possible solutions for the t-conorm was restricted to the cases with the minimum number of used sensors. The results of selected sensor networks are shown in Table 5 for the A1, A2, and A3 methods, as well as in Table 6 for the B1, B2, and B3 methods, adequately.

**Figure 14:**
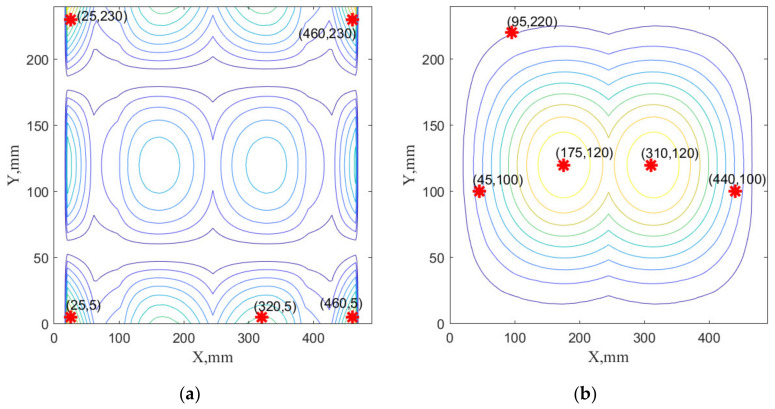
The sensor network SN#1 obtained using A1 method with 5 sensors in each direction and 2 dominated modes: the sensor network in X (**a**) and Y directions (**b**).

**Figure 15:**
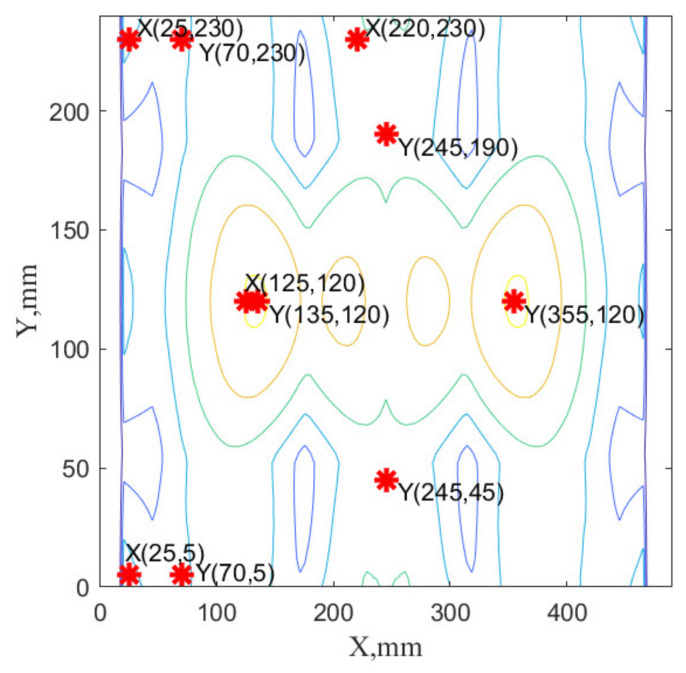
The sensor network SN#5 obtained using B2 method with 4 sensors in X direction and 5 sensors in Y direction obtained using three dominated modes. Labels X and Y indicate location for strain sensors oriented horizontally and vertically, respectively.

**Figure 16:**
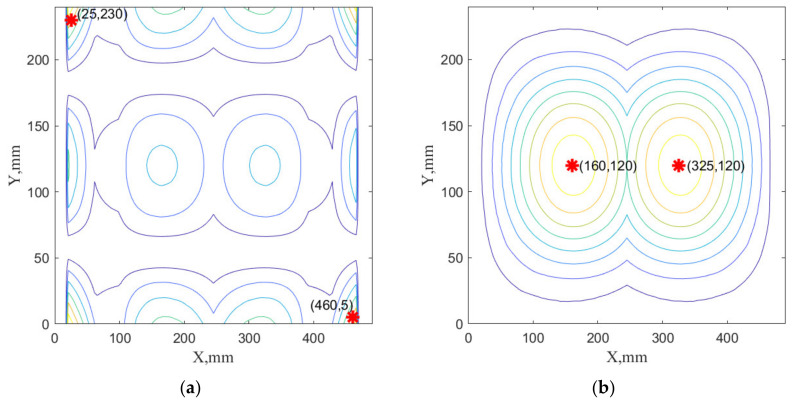
The sensor network SN#2R obtained using A2 method with 2 sensors in X direction and 2 sensors in Y direction obtained using two dominated modes: the sensor network in X (**a**) and Y directions (**b**).

The selected sensor networks specific for the considered cases are visualized in Figure 14, Figure 15 and Figure 16. They are only a graphical representation of the results shown in Table 3, Table 4, Table 5 and Table 6. The background of these figures shows the specific sum of the strains obtained for the number of modes in individual cases. In Figure 14 and Figure 16, sensor positions are shown separately to indicate that the sensor network comprises the composition of the optimal sensor network for the x-strain sensor and the y-strain sensors separately. In Figure 15, the sensor network is presented on one map to distinguish that the optimal sensor algorithm has used x-strains and y-strains commonly (methods B1, B2, and B3). To distinguish the sensors direction of the used in this case, X and Y labels were used in Figure 14.

## 4. Training of Machine Learning Algorithms

In the present study, two supervised learning schemes are considered for the performance evaluation of the OSP results. Five of the most typically employed machine learning algorithms, namely, k-NN, discriminant analysis, decision trees, Naïve Bayes, Support Vector Machines, are trained and tested for both learning schemes in a Matlab^®^ environment. The performance of classifiers for different sets of OSP is compared using error metrics and confusion matrices.

In the first learning scheme, acquired strain data were used to build binary classifier models establishing a description of normality representing undamaged conditions and abnormality indicating the presence of delamination damage in the composite structure. The output of classifiers is a discrete class label indicating the damage or undamaged state of the structure.

**Figure 17:**
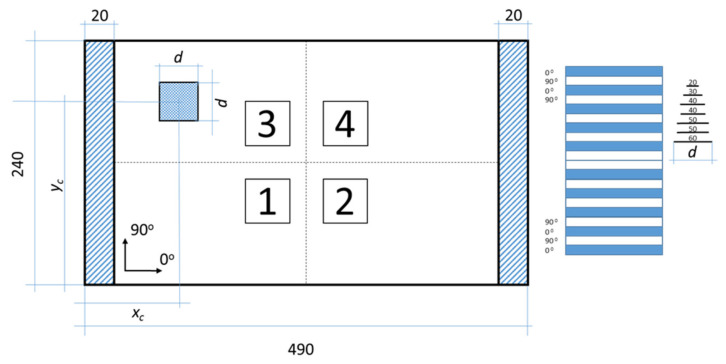
The schematic representation of the CFRP plate with possible locations of delamination.

In the second learning scheme, the training data consist of a set of strain data vectors together with known class labels that point to the geometrical location of the damage. In this study, the composite plate is divided into 4 substructures (zones) and the class labels are assigned according to the zone where the delamination damage is located (Figure 17). The output of the classifiers is a discrete class label that indicates the location of the damage in the structure. In this study, the size of a simulated damage remains constant (see Figure 17).

### 4.1. Preparation of Training Data

The plate FE model was built in the same way as described in Section 3.2, however, with a denser mesh of 90 × 24 elements. For the first supervised learning scheme, a modal analysis is performed for the first 7 modes of the developed model and the strain values in both main directions of the plate at the predefined sensor locations (Table 3, Table 4, Table 5 and Table 6) are recorded in a vector of 70 data points (7 modes × 10 sensors). A class label “0” is assigned to the vector representing the undamaged state of the plate.

In the next stage, artificial delamination damage simulating low-velocity impact to the plate is introduced to the FE model. The location and size of the delamination are varied for the training purposes of classifier models. The center point coordinates (*dx_c_* and *dy_c_*) of the delamination are selected according to the experiment plan (Table 7) developed to obtain the dynamic response of the plate for different damage scenarios. The depth and planar size *d* of the delamination are modelled according to the scheme depicted in Figure 17. For example, the planar size of the delamination between layers 2 and 3 is *d* = 20 mm and, in turn, *d* = 50 mm for the delamination between layers 8 and 9. Numerically, delamination is achieved by contact deactivation for the nodes in the area of delamination, while all other nodes between the corresponding layers are in contact. In total, 360 FE models were built and modal analysis was performed to determine strain values in both principal directions of the plate at predefined sensor locations (Table 3, Table 4, Table 5 and Table 6) for the first 7 modes. Therefore, for training purposes of machine learning algorithms, a matrix of 360 × 70 data points was obtained for all sensor networks.

For the first learning scheme, a class label “1” is assigned to all 360 data vectors that represent the damaged state of the plate. While for the second scheme, class labels “1” to “4” are designated corresponding to one of the four substructures on which the center point of the delamination damage is based (Table 7). Consequently, 360 sample points (90 data vectors for each of the 4 class labels representing the substructure of the location of the delamination damage in the plate) are acquired from the FE simulations for the second learning scheme and 361 sample points (1 for the undamaged state and 360 for damaged state) for the first scheme.

### 4.2. Data Pre-Processing for Training of Classification Models

One sample point includes a data vector of 70 sensor readings, 10 for each of the seven modes considered in the FE modal analysis. For instance, sensor network #1 comprises 5 sensor measurements in the *x* direction and the same number of readings in the *y* direction, while sensor network comprises #4–8 in *x* and 2 in *y* directions. To avoid a potential ill-conditioning problem caused by large differences in magnitude of strain values in different directions, normalizing all sensor readings with respect to the strain value of the first sensor in a respective direction is proposed. This task is also important considering the practical application of the method. It is of paramount interest to train classifiers for discriminant features that are independent of loading and environmental conditions and dependent only on the state of the structure.

### 4.3. Classification Models

In the first stage, five machine learning algorithms mentioned in the preamble of Section 4 were trained and tested for both learning schemes.

Normally, the larger the set of training samples, the better the classifier model, although the number of returns begins to decrease as soon as a certain amount of data is exceeded. Similarly, the larger the set of test samples, the more accurate the error estimates.

The parameters of the classifiers are optimized by using the validation of a classification model [58]. In this study, the *K*-fold cross-validation scheme was adopted to finetune the hyperparameters of the classifiers. In *K-fold* cross-validation, the data were randomly partitioned into *K* equal sets. For *K* number of folds, one set is reserved as validation data, while other *K* − 1 sets are used to train the model. The procedure is repeated *K* times so that every instance has been used exactly once for the validation of the *K* compact, trained models. The optimal number of *K* folds for cross-validation varies for different classifiers; therefore, in the initial phase, the standard approach of *K* = 10 folds was followed to find the best classifier for the defined learning schemes.

The performance of a classifier is typically evaluated by using error metrics and confusion matrices. For any classification problem, four outcomes are possible:

- True positives (*TP*)—the data are correctly classified as positive;
- True negatives (*TN*)—the data are correctly classified as negative;
- False positives (*FP*)—outcome is incorrectly classified as positive when it is actually negative;
- False negatives (*FN*)—outcome is incorrectly classified as negative when it is actually positive.

*TP* and *FP* are correct classifications, while *FP* and *FN* are incorrect. The overall accuracy of a classifier is calculated as follows.

(10) $$A=\frac{TP+TN}{TP+TN+FP+FN}\cdot 100\%$$

An effective method to evaluate the performance of a classifier is the definition of a confusion matrix. In this matrix, the rows correspond to the predicted class (Output Class), the columns show the true class (Target Class), while the cells located in matrix diagonal represent the percentage of the examples, which were correctly attributed to the defined classes. In fact, the cells in the matrix diagonal show the percentage of matching of true and predicted classes, while the other cells represent the percentage of cases mistakenly classified. The last column in the confusion matrix represents the accuracy of particular predicted classes, while the last row in this matrix represents the overall accuracy of classification. The perfect classification results were achieved when all the off-diagonal cells are zeros.

## 5. Results on Training and Testing of Classification Models

The present approach for the development of both learning schemes is described in detail based on the evaluation results of the predictive performance of the classifiers trained using the sensor network set #1. The description includes all three major steps of the classification process: training, cross-validation, and testing.

### 5.1. Training and Cross-Validation of Classification Models

To provide classifiers with large training and testing data, replicating the data points obtained by FE analysis multiple times and then adding random noise to the copied data for the generation of unique data points were proposed.

For the first learning scheme, the following procedure was adopted:

- The data vector that contains readings from the strain sensor for the undamaged state of the structure is replicated by a factor of 3600. Consequently, 3600 identical sample points are derived.
- Using Equation (11), random noise of 0.1% is added to all data points, which allows one to obtain 3600 unique sample points. A noise level of 0.1% allows obtaining unique sample points that are not equal to the original point yet very close to it in value; thus, the classifier can relate them to the same class as the original point:

(11) $$s=\overset{ˏ}{s}\left(1+\delta \left(2r-1\right)\right),$$

where $\overset{ˏ}{s}$ is a noise free data point, *r* is the uniformly distributed random values in the interval [0, 1], and *δ* is the noise level.

- Similarly, the procedure is performed for 360 data vectors containing strain sensor readings for the damaged state with only the difference being the multiplication factor, which is 10 in this case. As a result, in total, 7200 sample points (3600—for both the undamaged and damaged states of the plate) were acquired for the training data.
- The testing data comprise 720 sample points—360 original sample points for the damaged state and 1 original sample point for the undamaged state replicated 360 times using the procedure described above.

For the second learning scheme, testing data include only the original 360 sample points for the damaged state of the plate, while the training data of 3600 points were derived from the FE data using a multiplication factor of 10 and a noise level of 0.1%. It must be noted that there are no identical points among the training sample points and the testing data; hence, the latter are not used for training of the classifiers.

**Figure 18:**
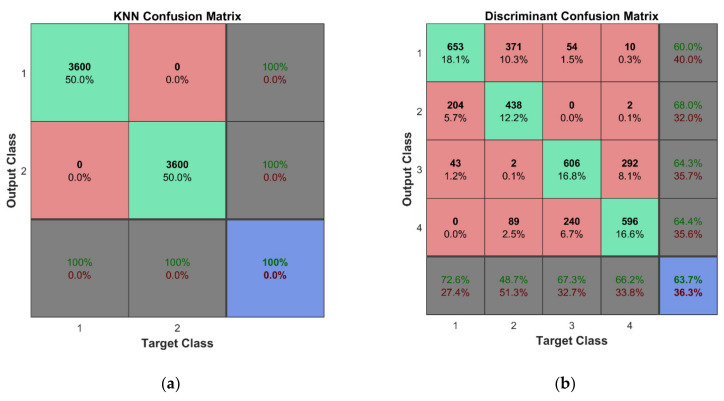
The obtained classification results for the best and worse classifiers: (**a**) k-NN and (**b**) discriminant analysis.

A ten-fold cross-validation scheme is used for both the training and cross-validation of the classifier models. The large set of the training data, ten folds for cross-validation, and fine-tuning of hyperparameters of the classifiers yielded almost perfect classification results for the first learning scheme. A classification accuracy of 100% was observed for four of five classifier methods. It should be noted that classifiers are trained for each mode separately; therefore, seven classification models were built for each of the five classifier methods considered in this study. In this manner, on the one hand, the chances of accidental misclassification are much lower as there are more results confirming estimated classes of observations, and on the other hand, it provides the possibility to select modes showing the best overall accuracy and to abandon the modes yielding unsatisfactory classification results if confirmed by more than one classifier. The confusion matrix for the k-NN classifier trained for the first mode is shown in Figure 18a. Similarly, confusion matrices with 100% overall accuracy are obtained for classification models of the other six modes.

For the second learning scheme, the overall accuracy of four out of five classifiers is above 97%; however, only k-NN and SVM methods could keep 100% performance. The discriminant analysis classifier shows relatively poor performance compared to the others; nevertheless, the results displayed are consistent (that is, the classification accuracy for all seven modes is approximately the same—65%). The confusion matrix for the discriminant classifier trained for the first mode is shown in Figure 18b. The overall summed accuracy of the classifier models is shown in Table 8.

### 5.2. Testing of Classification Models

**Figure 19:**
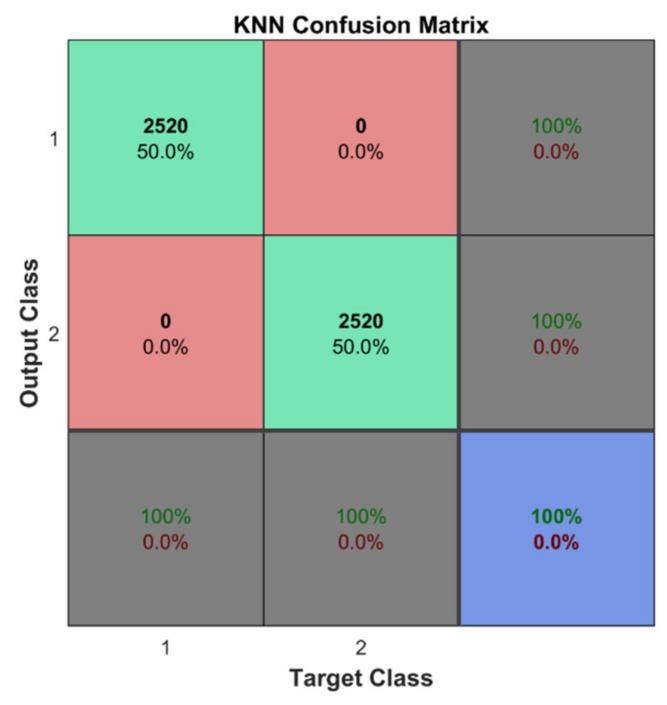
The obtained classification results for the k-NN classifier.

In addition, the predictive performance of the classifiers is investigated on unseen strain data. For the first learning scheme, the classifiers trained using 10-fold cross-validation were examined using the unseen testing data, which includes 360 cases for the undamaged state of the structure and the same number of cases for the plate with delamination damage located in different regions of the plate. The binary classification between the undamaged and damaged states of the composite plate was an easy task for four tested classifiers. The large set of training data and fine-tuning of the hyperparameters of the classifiers again yielded almost perfect classification. Confusion matrices with 100% overall accuracy as the one for the k-NN classifier provided in Figure 19 were obtained for four of five of the considered classifiers, with the exception of the discriminant analysis. This time, for the representation of the predictive performance of the classifiers, the confusion matrices for the seven classification models were summed in one plot. Thus, a total number of 2 × 360 × 7 class estimates was observed and included in the confusion matrix.

**Figure 20:**
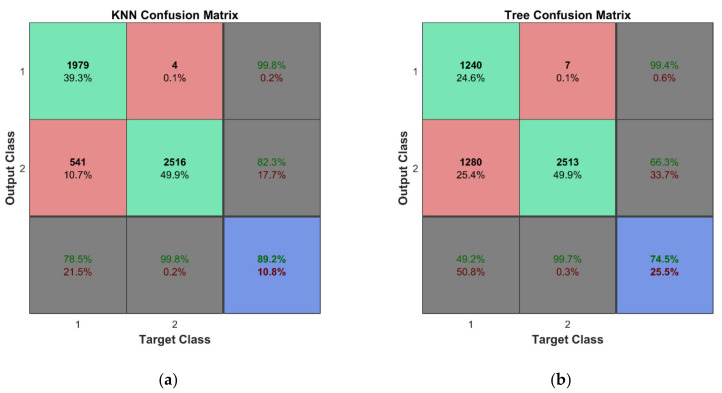
The results of classification for data with noise according to the first learning scheme for classifiers with the best performance: (**a**) k-NN and (**b**) decision trees.

In practice, measurement data are inevitably corrupted by noise; therefore, it was of interest to evaluate the predictive performance of classifiers on noisy sensor data. For this reason, the original testing data of 720 points were contaminated with noise of the following levels *δ* = 1%, 3%, and 5% using Equation (11). The results in the form of the overall accuracy of the classifier models are provided in Table 9. The confusion matrix for the two best performing classifier models (k-NN and decision trees) for *δ* = 1% is depicted in Figure 20. The results indicate effectiveness and robustness of the k-NN classifier to noisy measurement data up to the level of 5%, which is not typically exceeded by commonly employed strain sensors for SHM such as resistance strain gauges, fiber optic sensors, piezoelectric sensors, and micro-electromechanical systems (MEMS) sensors.

**Figure 21:**
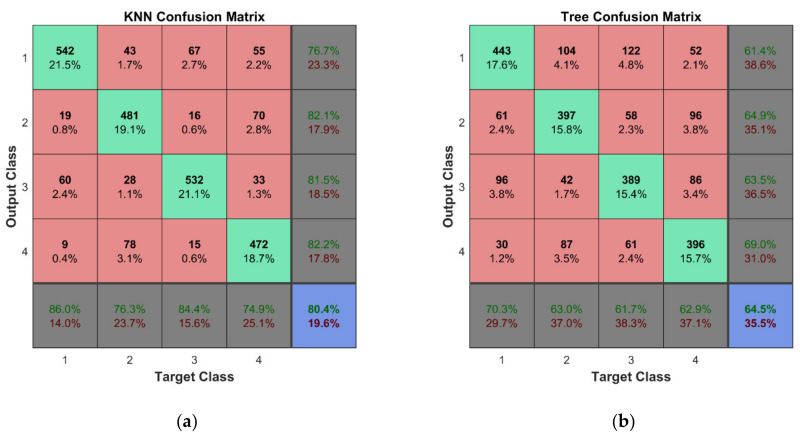
The results of classification for data with noise according to the second learning scheme for classifiers with the best performance: (**a**) k-NN and (**b**) decision trees.

The predictive performance of the classifiers for the second learning scheme is evaluated using a similar approach as for the first one. This time, the unseen testing data include only the 360 cases for the damaged state of the structure. The overall accuracy of the classifier models is shown in Table 10. The confusion matrices for the two best performing classifier models (k-NN and decision trees) for *δ* = 5% are shown in Figure 21. For the second classification problem, the k-NN algorithm shows outstanding performance—the classifier model is able to accomplish a task with an overall accuracy of 80.4% even for a noise level of 5%.

### 5.3. Performance Evaluation of Optimal Sensors Placement Results

The k-NN classifier is selected for the evaluation of the OSP results for both learning schemes. Classifier models are trained, cross-validated, and tested for each sensor network separately using the steps described in the previous section. The hyperparameters of the classifiers are optimized using a 10-fold cross-validation scheme. The evaluation results provided in Table 11, Table 12, Table 13 and Table 14 display the predictive performance of the classifiers on unseen testing data. The assumed notation in Table 11, Table 12, Table 13 and Table 14 is as follows: the notation “(4X,5Y) A2” means four sensors in the X direction and five sensors in the Y direction used by the A2 algorithm.

The results obtained show that sensor placements obtained using different methods have little effect on the accuracy of the binary classifier (Table 11 and Table 12) using the low noise levels. Larger differences among the sensor network results are observed only for the highest noise level. For the largest number of sensors (Table 11), the best accuracy is for the sensor network obtained using algorithm A2 (93.2% for 1% of the noise level). However, the difference in accuracy between the A2 and B2 algorithms becomes lower along with a higher level of noise. Finally, approach B2 is more robust relative to a higher level of noise, obtaining the highest accuracy for 5% of noise. In the case of a reduced number of sensors (Table 12), reasonable results can be obtained up to the noise level of 1%. In this case, approach B2 provides a more robust accuracy relative to the noise level.

The results obtained for the multiclass classification problem demonstrate that the number of sensors selected for algorithm training plays a significant role. The classification accuracy decreases rapidly as the number of sensors decreases. Comparing the classification results for the B1, B2, and B3 methods between Table 13 and Table 14, a significant decrease in accuracy can be observed in rows related to B1, B2, and B3 methods. This indicates that when the number of sensors is less than four, the results are no longer satisfactory. In addition to the selected optimization methods for approaches A1, A2, and A3 (shown in Table 13 and Table 14), they lead to satisfactory results in all cases. For a higher number of sensors, the best robustness to the noise level was achieved by the sensor networks obtained using the A2 and B3 approaches. The results for the multiclass classification show less accuracy to the similar level of noise in comparison to the binary classifiers.

### 5.4. Future Studies on Hardware Implementation of SHM System

**Figure 22:**
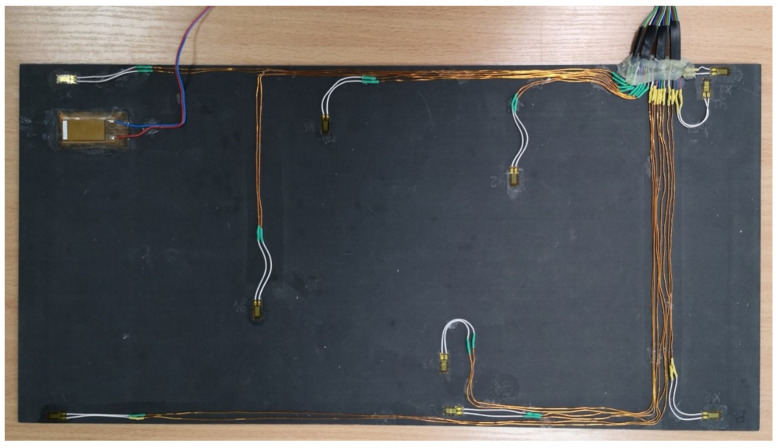
The exemplary CFRP plate with strain sensors network and a piezoelectric actuator.

The performed OSP studies will be useful for the planned hardware implementation of a SHM system demonstrator, which will be able to detect damage in composite plates based on the predefined optimized network of sensors placed on their surface. To date, initial studies on the development of such hardware implementation have already been done. The CFRP plate with identical properties, as was described in Section 2.1, was equipped with a network of HBM^®^ 1-CLY41 6/350ZE strain sensors (Darmstadt, Germany) and the MacroFiber Composite™ MFC P1 type (with d33 effect) piezoelectric actuator (Smart Materials, Sarasota, FL, USA) obtained based on the current investigation to test the performance of the measurement system (see Figure 22).

**Figure 23:**
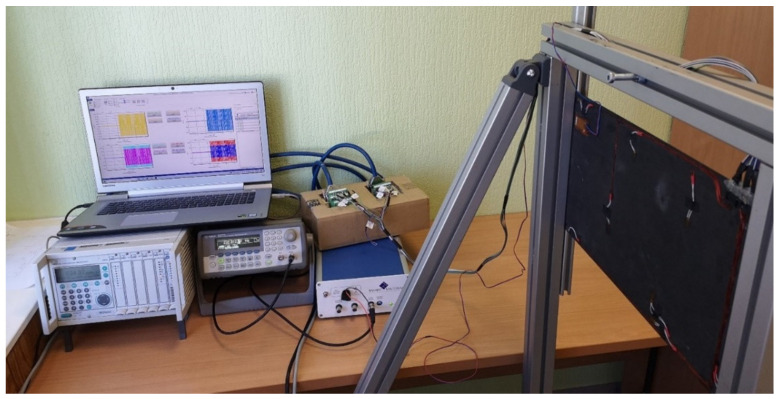
The experimental setup of hardware implementation of the developed SHM system.

The experimental setup consists of the Polytec^®^ PSV-500-3D scanning laser vibrometer used for the acquisition of the reference vibration response excited by the piezoelectric actuator, while dynamic strain measurements were performed using the experimental setup presented in Figure 23. It consists of the HBM^®^ MGCplus data acquisition system (Darmstadt, Germany) used for the acquisition of strain measurements using Catman dedicated software. The generation of the excitation signal was performed by the internal generator and amplified by the TREK PA05039 signal amplifier delivered by Smart Materials.

The initial tests were successful and allowed the confirmation of the possibility of identifying a simulated delamination in the CFRP plate prepared according to the parameters presented in Section 3, which provided a promising research direction for optimizing the performance of this system, particularly by applying the results of solution of the OSP problem investigated in this paper.

## 6. Discussion and Conclusions

This study presents results on the determination of OSP for the modal analysis of a composite plate with simulated damage within the developed SHM system. The results shown in this study are a part of an ongoing research study within the implemented project, which aims to develop and implement a fully functioning SHM system for the detection and identification of damage in composite structures. In this paper, the authors focused on the OSP problem, which is one of the initial stages of development of SHM system; however, it is a necessary step for the determination of the location of the sensors, which will be able to detect damage in an arbitrary position in the tested structure.

The obtained results show that detection and localization of the damaged state can be observed using especially selected sensor networks obtained using different OSP methods. This selection can be performed using the aggregation operator, which supports the designer of SHM system and provides cost reduction and high performance sensor network configuration.

The obtained results show that the detection is easily observed even for a small number of sensors. It also shows that the selection of the sensor network supported by the proposed aggregation t-conorm on the basis of different metrics provides satisfactory results, and the obtained sensor networks are very limited in the number of sensors used for the detection of the condition of the composite plate. Finally, it can also be observed that the robustness of the classification models to the noise level decreases along with the decreased number of sensors. A detailed discussion about the influence of noise level and the robustness of individual sensor network methods is presented in Section 5.3.

The localization of the delamination (multiclass classification problem) using the sensor networks also allows identifying the position of the delamination with high accuracy as long as the number of sensors is not very limited. It indicates that the proposed sensor network configuration based on the t-conorm should be taken from the center of the distribution of allowable sensor network configuration instead of the boundary with the lowest number of possible sensor numbers. The SHM system designer should be aware of that. It is worth it to emphasize that the localization of the delamination is less robust to the noise level in comparison to the detection problem, but the sensor networks in this research are derived on the basis of the modal matrix for healthy state of the composite plate only.

For the considered damage identification problem, the k-NN classifier shows the best performance in terms of overall classification accuracy. The selected ten-fold cross-validation scheme for finetuning classifier hyperparameters and the large set of the training data yielded almost perfect classification results for both learning schemes when noise-free testing data were used. Classification accuracy is over 97% is observed for four of five chosen classifier methods. Different results were observed for noisy testing data. The predictive performance of the classifiers decreases rapidly as the sensor readings are contaminated with noise. Only the k-NN classifier shows considerable noise resistance—the classification model is able to accomplish a task for a noise level of 5% with an overall accuracy of 77.1% and 80.4% for the first and second learning schemes, respectively. This indicates that commonly employed strain sensors such as resistance strain gauges, fiber optic sensors, piezoelectric sensors, and micro-electromechanical systems (MEMS) typically operating within the noise range of up to 5% are potential candidates for development of a sensor network for the SHM of composite structures.

The obtained OSP performance evaluation results show that sensor placement has little effect on the accuracy of the binary classifier, while classification accuracy for the multiclass classification problem decreases rapidly as the number of sensors decreases.

The results of OSP were applied to the developed SHM system, and the sensor configuration according to the obtained results was implemented in a real experiment. Preliminary hardware implementation was demonstrated in the paper, while systematic experiments and the validation of the developed SHM system will be the subject of the next studies by the authors. The developed SHM system combined with the proposed OSP method supported by FE models will allow the ability to locate sensors in an optimal manner even when possessing only the healthy condition of a tested structure.

## Figures and Tables

**Table 1:** The modes and corresponding natural frequencies (Hz) determined experimentally and numerically.

No.	Mode	EXP	FEM	Δ
1	(1,1)	24.80	25.27	1.89
2	(2,0)	57.81	57.96	0.26
3	(2,1)	78.91	77.57	1.70
4	(3,0)	158.2	159.95	1.11
5	(3,1)	179.49	177.45	1.14
6	(0,2)	209.57	211.49	0.92
7	(1,2)	215.82	217.54	0.80
8	(2,2)	247.46	243.01	1.80
9	(4,0)	309.18	308.86	0.10
10	(3,2)	319.73	314.43	1.66
11	(4,1)	330.18	330.03	0.04
12	(4,2)	446.29	432.99	2.98
13	(5,0)	508.2	521.19	2.56
14	(5,1)	533.59	535.47	0.35
15	(0,2)	580.47	586.34	1.01
16	(2,3)	606.05	591.33	2.43
Average				1.34

**Table 2:** The calibrated engineering constants for the CFRP plate.

Engineering constant	*E*_1_, GPa	*E*_2_, GPa	*G*_12_, GPa	*G*_23_, GPa	*ν*_12_, –
Calibrated value	136.85	7.60	3.4	7.32	0.348

**Table 3:** Sensor networks obtained using subsequently A1, A2, and A3 methods selected using t-norm maps.

SN #1											mX	mY	sX	sY
Sensor #	1	2	3	4	5	6	7	8	9	10	2	2	5	5
*x*, mm	25	25	320	460	460	45	95	175	310	440				
*y*, mm	5	230	5	230	5	100	220	120	120	100				
orientation	0	0	0	0	0	90	90	90	90	90				
**SN #2**														
Sensor #	1	2	3	4	5	6	7	8	9	10	4	3	4	5
*x*, mm	25	25	460	460	-	130	245	245	355	415				
*y*, mm	5	230	230	5	-	120	190	45	120	230				
orientation	0	0	0	0		90	90	90	90	90				
**SN #3**														
Sensor #	1	2	3	4	5	6	7	8	9	10	5	5	5	5
*x*, mm	25	140	140	345	465	55	55	225	225	430				
*y*, mm	120	230	5	230	5	140	15	20	200	95				
orientation	0	0	0	0	0	90	90	90	90	90				

**Table 4:** Sensor networks obtained using subsequently B1, B2, and B3 methods selected using t-norm maps.

SN #4											nM	nS
Sensor #	1	2	3	4	5	6	7	8	9	10	2	10
*x*, mm	25	25	165	165	320	320	460	460	175	310		
*y*, mm	5	230	230	5	230	5	230	5	125	125		
orientation	0	0	0	0	0	0	0	0	90	90		
**SN #5**												
Sensor #	1	2	3	4	5	6	7	8	9	10	3	10
*x*, mm	25	25	125	220	70	70	245	245	355	135		
*y*, mm	5	230	120	230	5	230	190	45	120	120		
orientation	0	0	0	0	90	90	90	90	90	90		
**SN #6**												
Sensor #	1	2	3	4	5	6	7	8	9	10	2	10
*x*, mm	25	85	90	135	245	245	355	390	410	465		
*y*, mm	100	5	230	120	200	40	115	230	5	100		
orientation	90	90	90	90	90	90	90	90	90	90		

**Table 5:** Sensor networks obtained using subsequently A1, A2, and A3 methods selected using t-conorm maps.

SN #1R					mX	mY	sX	sY
Sensor #	1	2	3	4	2	2	2	2
*x*, mm	25	460	175	310				
*y*, mm	230	5	120	120				
orientation	0	0	90	90				
**SN #2R**								
Sensor #	1	2	3	4	2	2	2	2
*x*, mm	25	460	160	325				
*y*, mm	230	5	120	120				
orientation	0	0	90	90				
**SN #3R**								
Sensor #	1	2	3	4	2	2	2	2
*x*, mm	25	465	245	245				
*y*, mm	230	5	200	40				
orientation	0	0	90	90				

**Table 6:** Sensor networks obtained using subsequently B1, B2, and B3 methods selected using t-conorm maps.

	SN #4R		nM	nS	SN #5R		nM	nS	SN #6R		nM	nS
Sensor #	1	2	2	2	1	2	2	2	1	2	2	2
*x*, mm	175	310			25	160			245	245		
*y*, mm	120	120			230	120			200	40		
orientation	90	90			0	90			90	90		

**Table 7:** The experimental plan for the considered damage scenarios.

No.	*dx_c_*	*dy_c_*	Class
1	55	35	1
2	55	45	1
…			
10	55	125	3
…			
199	275	35	2
…			
360	435	205	4
361	–	–	0

**Table 8:** The results of classification for all considered classifiers.

Classifier	1st Learning Scheme	2nd Learning Scheme
k-NN	100%	100%
Discriminant analysis	74.9%	65.9%
Decision trees	100%	99.1%
Naïve Bayes	100%	97.2%
Support Vector Machines	100%	100%

**Table 9:** The influence of noise on classification performance for the first learning scheme.

Classifier	Noise Level			
	0%	1%	3%	5%
k-NN	100%	89.2%	83.6%	77.1%
Discriminant analysis	75.7%	66.2%	58.9%	54.7%
Decision trees	100%	74.5%	69.4%	67.9%
Naïve Bayes	100%	64.9%	60.1%	56.5%
Support Vector Machines	100%	67.5%	53.2%	50.4%

**Table 10:** The influence of noise on classification performance for the second learning scheme.

Classifier	Noise Level			
	0%	1%	3%	5%
k-NN	100%	95.1%	86.1%	80.4%
Discriminant analysis	68.0%	55.9%	45.9%	39.2%
Decision trees	99.7%	86.3%	71.9%	64.5%
Naïve Bayes	98.0%	75.7%	57.4%	50.0%
Support Vector Machines	100%	77.8%	59.1%	54.2%

**Table 11:** OSP performance for the first learning scheme (SN #1–#6).

Sensor Network	Noise Level			
	0%	1%	3%	5%
1 (5X,5Y) A1	100%	89.2%	83.6%	77.1%
2 (4X,5Y) A2	100%	93.2%	85.2%	77.7%
3 (5X,5Y) A3	100%	91.7%	74.8%	68.4%
4 (5X,5Y) B1	100%	88.1%	75.8%	69.3%
5 (5X,5Y) B2	100%	89.0%	82.5%	78.2%
6 (5X,5Y) B3	100%	88.9%	79.7%	72.6%

**Table 12:** OSP performance for the first learning scheme (SN #1R–#6R).

Sensor Network R	Noise Level			
	0%	1%	3%	5%
1(2X,2YR) A1	100%	83.0%	74.5%	63.9%
2(2X,2YR) A2	99.8%	75.9%	67.1%	64.4%
3(2X,2YR) A3	99.9%	80.3%	66.1%	57.8%
4(0X,2YR) B1	99.7%	78.4%	67.2%	61.5%
5(1X,1YR) B2	98.8%	74.8%	68.2%	67.3%
6(0X,2YR) B3	98.9%	70.7%	63.6%	59.6%

**Table 13:** OSP performance for the second learning scheme (SN #1–#6).

Sensor Network	Noise Level			
	0%	1%	3%	5%
1 (5X,5Y) A1	100%	95.1%	86.1%	80.4%
2 (4X,5Y) A2	100%	97.4%	88.8%	82.9%
3 (5X,5Y) A3	100%	96.1%	85.9%	78.3%
4 (5X,5Y) B1	100%	95.8%	81.0%	71.6%
5 (5X,5Y) B2	99.7%	94.0%	85.9%	80.5%
6 (5X,5Y) B3	100%	95.2%	88.0%	83.7%

**Table 14:** OSP performance for the second learning scheme (SN #1R–#6R).

Sensor Network R	Noise Level			
	0%	1%	3%	5%
1(2X,2Y) A1	97.1%	79.6%	68.5%	61.7%
2(2X,2Y) A2	96.7%	79.4%	68.5%	60.7%
3(2X,2Y) A3	93.8%	71.0%	55.8%	48.0%
4(0X,2Y) B1	50.1%	42.9%	37.4%	38.5%
5(1X,1Y) B2	80.0%	54.6%	41.3%	38.9%
6(0X,2Y) B3	47.3%	39.0%	35.2%	33.2%

## Data Availability

The data that support the findings of this study are available from the first author, Sandris Ručevskis, upon reasonable request.
